# Age-Related Increase of Collagen/Fibrin Deposition and High PAI-1 Production in Human Nasal Polyps

**DOI:** 10.3389/fphar.2022.845324

**Published:** 2022-05-31

**Authors:** Ara Jo, Tae Gyu Choi, Jung Yeon Han, Mark H. Tabor, Narasaiah Kolliputi, Richard F. Lockey, Seong H. Cho

**Affiliations:** ^1^ Division of Allergy-Immunology, Department of Internal Medicine, Morsani College of Medicine, University of South Florida, Tampa, FL, United States; ^2^ Department of Biochemistry and Molecular Biology, School of Medicine, Kyung Hee University, Seoul, South Korea; ^3^ Department of Otolaryngology-Head and Neck Surgery, Morsani College of Medicine, University of South Florida, Tampa, FL, United States; ^4^ Division of Allergy-Immunology, James A. Haley Veterans’ Hospital, Tampa, FL, United States

**Keywords:** nasal polyps, aging, tissue remodeling, submucosal glands, antimicrobial peptides and proteins

## Abstract

**Objective:** Our previous studies showed an age-related increased prevalence of nasal polyps (NP) and reduced production of S100A8/9 in elderly patients with chronic rhinosinusitis with NP (CRSwNP). In this study, we investigated an unbiased age-related gene expression profile in CRSwNP subjects and healthy controls, and further identified the differences in their tissue remodeling.

**Methods:** Microarrays using NP and uncinate tissues from health controls (elderly, age ≥65 vs. non-elderly, age 18–49) were performed, and differentially regulated genes were analyzed. Quantitative real-time PCR (qPCR), Immunostaining, Periodic acid-Schiff (PAS), trichrome staining, Western blot, and ELISA were performed for further investigation.

**Results:** Microarrays identified differentially expressed genes according to disease and age; 278 in NP vs. controls, 75 in non-elderly NP vs. non-elderly controls, and 32 in elderly NP vs. elderly controls. qPCR confirmed that the PLAT gene was downregulated and the SERPINB2 gene upregulated in NP vs. controls. The serous glandular cell-derived antimicrobial protein/peptide-related genes such as BPIFB3, BPIFB2, LPO, and MUC7 were remarkably reduced in NP, regardless of age. SERPINE1 gene (plasminogen activator inhibitor-1, PAI-1) expression was significantly increased in elderly NP versus elderly controls. IHC and western blot confirmed significantly decreased production of MUC7 and LPO in NP versus controls. There was a trend of age-related reduction of submucosal gland cells in normal controls. Trichrome and immunofluorescence staining demonstrated an age-related increase of collagen and fibrin deposition in NP, consistent with increased PAI-1 production.

**Conclusion:** This study demonstrated age-related differential glandular remodeling patterns and fibrosis in NP and normal controls. PAI-1 expression was significantly increased in elderly NP versus elderly controls, suggesting PAI-1 as a potential treatment target in elderly NP.

## Background

Chronic rhinosinusitis (CRS) is a prevalent chronic rhinologic disease, characterized by persistent symptomatic inflammation of the nasal mucosa and paranasal sinuses. It is classified as CRS with nasal polyps (CRSwNP) or without nasal polyps (CRSsNP), based on the presence of polyps. NPs are edematous masses of inflamed mucosa and usually originate from the ostiomeatal complex in and around the middle nasal meatus. They are characterized by inflammatory cell infiltration and abnormal tissue remodeling (Stevens et al., 2014; de Loos et al., 2019; Fokkens et al., 2020). However, the mechanisms and the pathologic process responsible for NP are not fully understood.

Submucosal glands (SMG) consist of mucous and serous cells and contribute to mucus hypersecretion in airway inflammation by secreting mucins, glycoproteins, ions, and water. Serous cells produce and secrete antimicrobial peptides and proteins (AMP), which are important components of the innate immune response, while mucous cells are a source of gel-forming mucins (Widdicombe and Wine, 2015; Ma et al., 2018). Production of some AMP was reduced compared to normal tissue in NP tissues, which may reflect a decrease in SMG (Seshadri et al., 2012). Mucins are large heterogeneous macromolecules which are encoded by MUC genes and are produced by epithelial goblet cells and SMG (Groneberg et al., 2003). The antimicrobial-rich mucus gel is formed by mucins, and mucins can exert protective functions by adhesion to the surface of microorganisms (Situ and Bobek, 2000; Linden et al., 2008). The secreted non-gel-forming mucin, MUC7, an AMP, is primarily expressed in serous cells of SMG (Sharma et al., 1998). Similar distribution of other MUC genes is found in NP (Geiszt et al., 2003; Ali et al., 2005; Martínez-Antón et al., 2006). Lactoperoxidase (LPO) is secreted primarily by serous cells of the respiratory submucosa and has airway antibacterial activity and can promote bacterial clearance (Gerson et al., 2000; Wijkstrom-Frei et al., 2003). The BPI fold-containing family (BPIF) gene encodes palate, lung, and nasal epithelium carcinoma associated proteins (PLUNC) and members of the BPI-PLUNC family act as AMP and are expressed in human airway epithelium and SMG (Gakhar et al., 2010). Other reports indicate that the expression of PLUNCs is decreased in NP, and this decreased expression is indicative of a reduced number of SMG in NP (Seshadri et al., 2012; Wei et al., 2014).

The fibrinolytic system consists of a number of proteases and protease inhibitors that regulate the conversion of plasminogen to its active form, plasmin. Fibrinolysis is regulated by plasminogen activator inhibitor-1 (PAI-1; SERPINE1) and PAI-2 (SERPINB2), which inhibit the proteolytic activity of two major plasminogen activators, tissue-type (tPA; PLAT) and urokinase-type (uPA; PLAU). The fibrinolysis system breaks down the extracellular matrix and controls cell adhesion and migration and thus participates in tissue remodeling. Previous studies report excessive fibrin deposition caused by decreased fibrinolysis activity and increased expression of coagulation factors in human NP (Takabayashi et al., 2013; Takabayashi and Schleimer, 2020).

Aging is a major risk factor for many chronic diseases, follows defined and important biological processes, and is a particularly influential factor in CRSwNP. The prevalence of CRSwNP is increased after age 50. Several genes associated with inflammatory mechanisms and fibrotic changes have been identified in NP. We previously reported that there is a significant age-related increased prevalence of NP and reduced production of an AMP, calprotectin (S100A8/9), in elderly NP (Cho et al., 2012; Cho et al., 2015). Several studies indicate that SERPINE1 expression is not only elevated in the elderly but also significantly enhanced in various clinical conditions (De Taeye et al., 2005; Yamamoto et al., 2005; Cesari et al., 2010; Ghosh et al., 2010). However, the age-related changes in the gene expression profile in CRSwNP remain unclear. Microarray technology is an established unbiased approach to measure gene expression values in various diseases, i.e., CRS (Plager et al., 2010; Böscke et al., 2017; Tyler et al., 2017), asthma (Bigler et al., 2017; Singhania et al., 2018), COPD (Boudewijn et al., 2017; Willis-Owen et al., 2018), and acute respiratory infections (Henrickson et al., 2018). This is a useful tool to monitor the age-related changes in gene expression in NP and normal controls.

The purpose of this study is to identify genes that are differentially expressed with CRS disease, age or both. This approach can provide an unbiased identification of new biomarkers of age-related differences in the pathogenesis of NP. In this study, we report significant age-related differences in the production of tissue remodeling-related markers in NP and normal controls.

## Materials and Methods

### Patients and Gene Expression Datasets

Sinonasal tissues were obtained during sinus surgeries performed at the University of South Florida (USF) and Northwestern University. For the microarray, NP tissues and uncinate tissues from normal healthy controls were divided into elderly (≥65 years old), or non-elderly (18–49 years old) according to age. There was a 15 years age gap between the two age groups to make a clear age-related distinction of various findings. Supplementary Tables SE1, SE2 show the subject numbers and characteristics of the NP patients and normal controls, showing similar age, sex, and racial distribution except higher prevalence of atopy and asthma in NP patients.

This study protocol was approved by the institutional review boards (IRB) at the University of South Florida (USF) and Northwestern University. Written informed consent was obtained from all study subjects before they participated in the study. All experiments were performed in accordance with relevant guidelines and regulations.

### RNA Preparation and Microarray

For microarray and qPCR analysis, total RNA was isolated from NP tissues and control uncinate tissues. RNA concentration was calculated, and the highest quality RNA used for microarray and real-time PCR analysis. The quality of total RNA was evaluated by Bioanalyzer (Agilent Technologies, Santa Clara, CA, United States). The samples were at a concentration ≥125 ng/μl and a A260/A280 ratio that was ≥1.80 and a RIN value that was ≥7.0 were considered acceptable. The highest quality RNA was used to generate labeled cRNA and hybridized to Affymetrix Human Gene 2.1ST array following the manufacturer’s protocol (Affymetrix Inc., Santa Clara, CA, United States). Chip processing and image capturing were performed using Affymetrix AGCC software.

### Microarray Validation by Real-Time PCR

Real-time PCR (qPCR) was used for validation and confirmation of gene expression results obtained from the microarray analysis. For analysis of MUC7 (Hs00379529_m1), LPO (Hs00976400_m1), PLAT (Hs00263492_m1), SERPINE1 (Hs00167155_m1), SERPINB2 (Hs01010736_m1), and GAPDH (Hs02786624_g1), primers were purchased for Taqman assays (Applied Biosystems/Thermo Fisher Scientific, Waltham, MA, United States). Expression of GAPDH was used as the normalizing gene and relative quantitation was calculated using the comparative 2^−ΔΔCT^ method.

### Histologic and Immunofluorescence Analysis

Sinonasal tissue sections were stained with periodic acid-Schiff (PAS) to observe serous and mucous glands. For immunohistochemistry staining, sinonasal tissue slides were deparaffinized with xylene and rehydrated with gradually decreased ethanol in deionized water. Heat mediated antigen retravel was performed in a citrate buffer (pH 6.0) using microwave for 20 min. The ImmPRESS Duet Double Staining Polymer Kit (Vector Laboratories, Burlingame, CA, United States) was used following the manufacturer’s protocol. Slides were blocked with 2.5% normal horse serum for 20 min at room temperature, followed by primary antibodies, mouse monoclonal anti-α smooth muscle actin (diluted 1:500; Cell Signaling, Danvers, MA, United States) and rabbit polyclonal anti-MUC7 (diluted 1:500; Invitrogen, Waltham, MA, United States) or rabbit polyclonal anti-LPO (diluted 1:500; Novus, Centennial, CO, United States) overnight at 4°C. Optimal detection with provided DAB substrate (brown color) was performed first, then applied red substrate (AP) until desired intensity developed. Slides were dehydrated with ethanol and cleared with xylene followed by mounting with coverslip using permount mounting medium (Fisher Scientific, Hampton, NH, United States). Images were taken using a digital light microscope (Keyence, Osaka, Japan). MUC7 and LPO positive stain was separated from αSMA positive stain by Color Deconvolution using ImageJ software. Quantification of integrated density by threshold was also performed on ImageJ (National Institutes of Health, Bethesda, MD, United States). αSMA was stained to optimize glandular structure. Trichrome staining was used to evaluate collagen deposition and trichrome positive areas were quantified using ImageJ software.

Deparaffinized and rehydrated sinonasal tissue slides were incubated with 1% BSA for 1 h at room temperature, followed by incubation with a monoclonal anti-fibrin antibody (diluted 1: 500; GeneTex, Irvine, CA, United States) overnight at 4°C. Slides were then washed and incubated with Alexa-fluor 488 (diluted 1:1,000; Invitrogen, Carlsbad, CA, United States) for 2 h. Whole slides were mounted using Fluoroshield mounting medium containing DAPI (Abcam, Cambridge, MA, United States) after washing. Immunofluorescence was visualized using confocal laser microscopy (Olympus FV1200, Tokyo, Japan). The intensity of immunofluorescence was quantified by measuring the integrated density using the ImageJ software.

### Western Blot

Western blot analyses were performed using sinonasal tissue extracts, prepared from samples collected during sinus surgeries at USF. For immunoblotting, the proteins were separated on 8%–12% SDS-PAGE and transferred onto nitrocellulose membranes (Pall Corporation, Washington, NY, United States). After blocking, primary antibodies (anti-MUC7 and anti-LPO) were diluted according to the manufacturer’s instructions. The blotted proteins were detected with an enhanced chemiluminescence (ECL) detection system (Thermo Scientific Pierce, Rockford, IL, United States). GAPDH was used as a loading control.

### ELISA

The PAI-1 concentrations in sinonasal tissue extracts were measured in duplicate using commercially available ELISA kits (Assaypro, St Charles, MO, United States). All procedures followed the manufacturer’s instructions. The protein levels in the tissue homogenates were normalized to the concentration of total protein. The minimum detectable concentration was 78 pg/ml for human PAI-1.

### Statistical Analysis

Microarray data and resultant heatmap plots were analyzed using BRB-Array Tools Version 4.6 (https://brb.nci.nih.gov/BRB-ArrayTools/index.html) (Simon et al., 2007). All other statistical analyses were accomplished in the R language environment (http://www.r-project.org) using Statistical Package for Social Sciences (SPSS) software (version 25, SPSS Inc., Chicago, IL, United States). All other results were expressed as mean ± SEM. Statistical differences were determined by the unpaired Student’s t-test using the GraphPad statistical software (GraphPad Software Inc., La Jolla, CA, United States). In all statistical analyses, a *p* value of ≤ 0.05 was considered significant.

## Results

### Identification of Differentially Expressed Genes Between Nasal Polyp Subjects Versus Normal Controls

**FIGURE 1:**
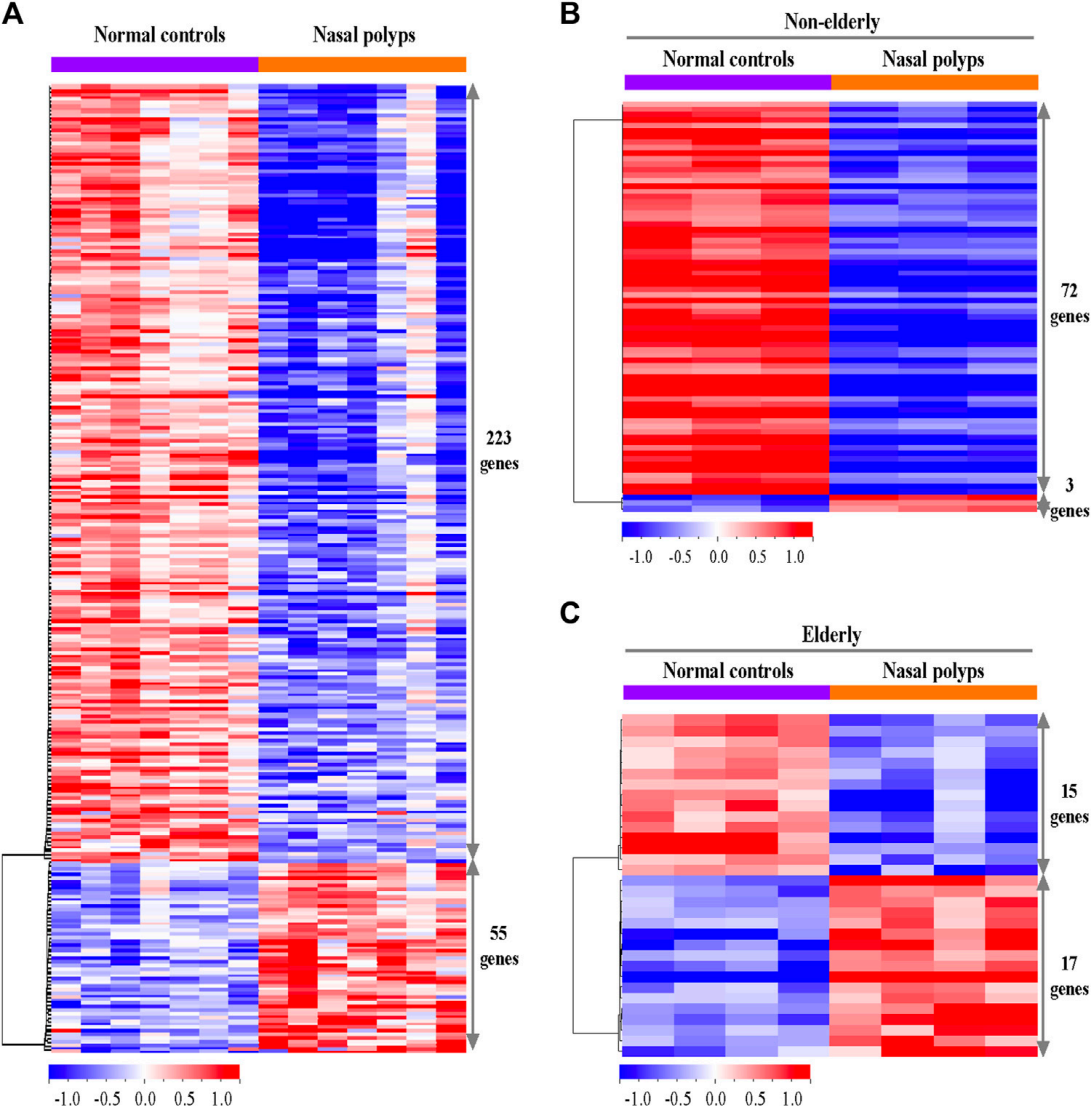
Differentially expressed genes in the sinonasal tissues from subjects with NP and normal controls. **(A)** The heatmaps represent expression profiles of the 278 differentially expressed genes in normal controls versus NP, **(B)** the 75 differentially expressed genes in non-elderly normal controls versus non-elderly and **(C)** the 32 differentially expressed genes in elderly normal controls versus elderly NP. A red-blue color scale depicts expression levels (upregulated; red and downregulated; blue).

The microarray dataset (*n* = 14) were utilized to identify the differentially expressed genes (DEGs) of sinonasal tissues between normal versus NP subjects. The 26,401 probe sets annotated for unique genes, and micro RNAs (miRNAs) were filtered by at least two absolute values of the log2 scale, representing the same gene expression level. After filtering for probe set intensity, 3,433 probe sets were analyzed, using a univariate statistical algorithm of class comparison (*p*-values set to 0.001 as the significant threshold). Two hundred and seventy-eight unique genes were obtained of which 55 were upregulated and 223 downregulated in NP versus controls (Figure 1A). Most of the differentially regulated genes were downregulated (80.22%) while 19.78% were upregulated in the NP. Among the DEGs, the top-ranked 15 upregulated and downregulated genes between NP subjects versus normal controls are listed in Table 1. The upregulated genes include fibrinolytic system (e.g., SERPINB2), cell adhesion and microbial infection (e.g., carcinoembryonic antigen-related cell adhesion molecule 5 (CEACAM5), macrophage (e.g., CD68) and T2-associated gene expression marker (e.g., POSTN). Downregulated genes include antimicrobial activity-related genes (e.g., BPIFB2, BPIFB3, LPO, and MUC7) and fibrinolytic system (e.g., PLAT).

**TABLE 1:** List of upregulated and downregulated genes.

Top upregulated genes	FDR	Fold change	*p*-value	Top downregulated genes	FDR	Fold change	*p*-value
Nasal polyps versus Normal controls							
CEACAM5	0.00054	10.24	3.22E-05	SCGB2A2	0.0010	−21.77	1.81E-04
UPK1B	0.00196	6.75	1.58E-03	PIP	0.0025	−19.55	2.43E-03
SERPINB2	0.00249	5.81	2.45E-03	BPIFB3	0.0014	−19.50	6.90E-04
PTHLH	0.00191	3.67	1.53E-03	BPIFB2	0.0017	−18.64	1.15E-03
RNASE2	0.00238	3.38	2.28E-03	GLYATL2	0.0016	−14.42	1.08E-03
CDH26	0.00145	3.35	8.20E-04	LPO	0.0027	−12.82	2.65E-03
C1QC	0.00141	3.07	7.32E-04	HP	0.0017	−11.69	1.14E-03
CD68	0.00118	3.04	4.12E-04	IBSP	0.0018	−11.77	1.33E-03
APOC1	0.00093	2.75	1.23E-04	F5	0.0012	−9.79	3.99E-04
PTPRQ	0.00242	2.69	2.36E-03	CLDN10	0.0010	−9.53	1.74E-04
ALOX15	0.00235	2.67	2.23E-03	MUC7	0.0011	−9.44	3.18E-04
POSTN	0.00231	2.52	2.16E-03	AZGP1	0.0016	−6.71	1.02E-03
C1QB	0.00191	2.51	1.53E-03	KCNN4	0.0013	−5.75	5.86E-04
LAMB3	0.00101	2.45	2.04E-04	CA2	0.0014	−5.28	8.07E-04
LOC401585	0.00116	2.41	3.72E-04	SLC5A1	0.0027	−5.17	2.65E-03
Non-elderly nasal polyps versus Non-elderly normal controls							
SLC9A3	0.042	4.91	8.60E-04	C6orf58	0.02	−80.10	1.24E-05
LY6E	0.042	2.47	9.56E-04	CRISP3	0.035	−76.82	1.89E-04
UCP2	0.042	2.46	6.49E-04	PRB3	0.042	−75.64	6.13E-04
				PIP	0.035	−67.33	2.08E-04
				BPIFB2	0.035	−59.81	2.61E-04
				LPO	0.035	−38.65	8.80E-05
				CLDN10	0.035	−25.64	1.11E-04
				F5	0.035	−17.00	2.69E-04
				AZGP1	0.035	−13.78	2.51E-04
				SLC5A1	0.035	−13.28	2.63E-04
Elderly nasal polyps versus Elderly normal controls							
CEACAM5	0.003	12.23	1.70E-06	SEC14L3	0.038	−5.04	1.57E-04
PTHLH	0.059	4.35	4.38E-04	LOC102723344	0.075	−3.34	8.95E-04
SNORD114-20	0.038	4.11	1.34E-04	LINC01208	0.038	−3.19	2.06E-04
SERPINE1	0.038	3.67	1.44E-04	MIR4500	0.065	−3.04	5.64E-04
FAM83D	0.012	3.13	1.84E-05	SPP1	0.049	−2.57	3.10E-04
APOC1	0.075	2.96	8.63E-04	LOC100506682	0.011	−2.49	1.27E-05
CD68	0.054	2.88	3.64E-04	GRIK2	0.003	−2.29	2.60E-06
GPNMB	0.038	2.44	9.52E-05	ACSS3	0.075	−2.24	8.04E-04
PLVAP	0.038	2.09	1.75E-04	HSD17B7	0.038	−2.10	2.07E-04
GJA1	0.049	2.07	2.95E-04	AR	0.065	−2.06	5.68E-04

The differential gene expression between non-elderly NP subjects and non-elderly normal controls and elderly NP subjects and elderly normal controls was investigated to understand age-related differences in NP-associated gene regulations. Seventy-five significant genes were identified when comparing the DEG between non-elderly NP subjects versus non-elderly normal controls. Among them, 3 genes (4.0%) are upregulated and 72 (96.0%) downregulated (Figure 1B). The top 3 upregulated and 10 downregulated genes are listed in Table 1. The antimicrobial activity-related genes (e.g., BPIFB2 and LPO) were downregulated. Thirty-two of unique genes were identified when comparing the DEG between elderly NP subjects versus normal elderly controls. Seventeen genes (53%) are upregulated, and 15 genes (47%) downregulated (Figure 1C). Among the DEGs, the top 10 upregulated and downregulated genes are listed in Table1. These upregulated genes include fibrinolytic system (e.g., SERPINE1), macrophage marker (e.g., CD68), and cell adhesion and microbial infection marker (e.g., CEACAM5).

### Histopathologic Changes of Submucosal Glands in Subjects With Nasal Polyps and Normal Controls

**FIGURE 2:**
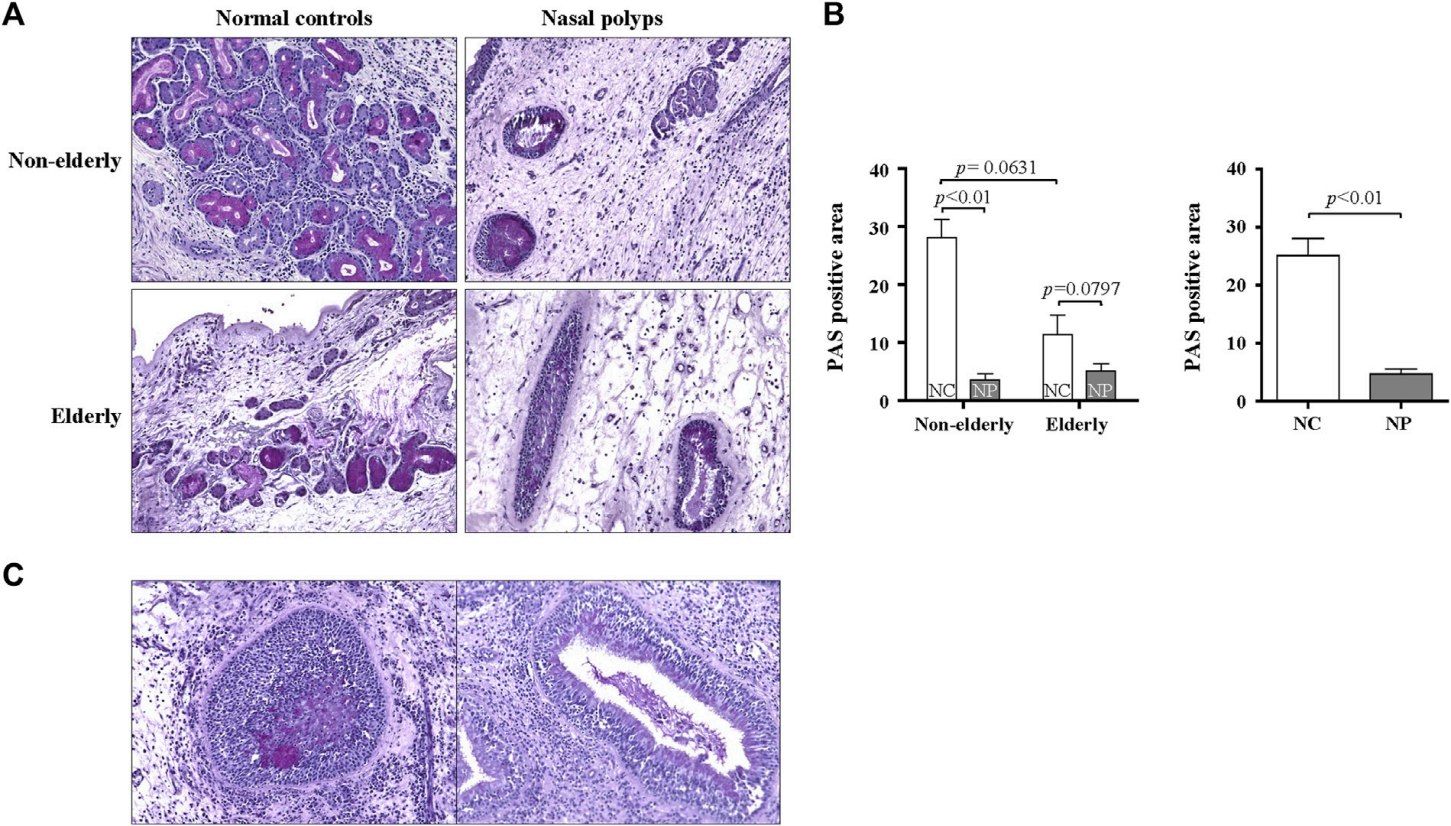
Histopathologic changes of the submucosal gland in the sinonasal tissues from subjects with NP and normal controls. **(A)** Representative PAS staining for histopathologic changes on sinonasal tissue sections of NP and normal controls. Original magnification ×200. **(B)** Quantification of the PAS positive areas was performed using ImageJ. Data are expressed as the means ± SE (non-elderly NC versus elderly NC, *n* = 19, *p* = 0.0631; NC versus NP, *n* = 30, *p* < 0.01). NC, normal control; NP, nasal polyp. **(C)** PAS stained abnormal gland morphology. Original magnification ×200.

Genes related to tissue remodeling, such as submucosal glandular remodeling and fibrinolysis, are highly downregulated or upregulated in the microarray. Therefore, PAS staining was utilized to determine the distribution and morphology of serous and mucous cells of SMG. There was a trend of age-related decrease of PAS-positive SMG area in normal controls (Figures 2A,B, *n* = 19, *p* = 0.0631). Relative glandular metaplasia was observed in the elderly controls compared with the non-elderly (Figure 2A). Additionally, there was significant reduction of PAS positive glandular area in non-elderly NP compared with non-elderly normal control (Figure 2B, *n* = 30, *p* < 0.01), and overall NP compared with normal control (Figure 2B, *n* = 48, *p* < 0.01). On the other hand, there was no significant difference in positive glandular area between elderly NP versus elderly normal control (Figure 2B, *n* = 18, *p* = 0.0797). The ductal metaplasia including cystically dilated gland was present in elderly control tissues and even more prominent in NP tissues (Figure 2A). Interestingly, abnormal gland morphology such as multilayered squamous metaplasia are observed in NP (Figure 2C). There was no significant age-related histologic difference in glandular remodeling patterns between elderly NP versus non-elderly NP.

### Differential Expression of MUC7 and Lactoperoxidase in Subjects With Nasal Polyps and Normal Controls

**FIGURE 3:**
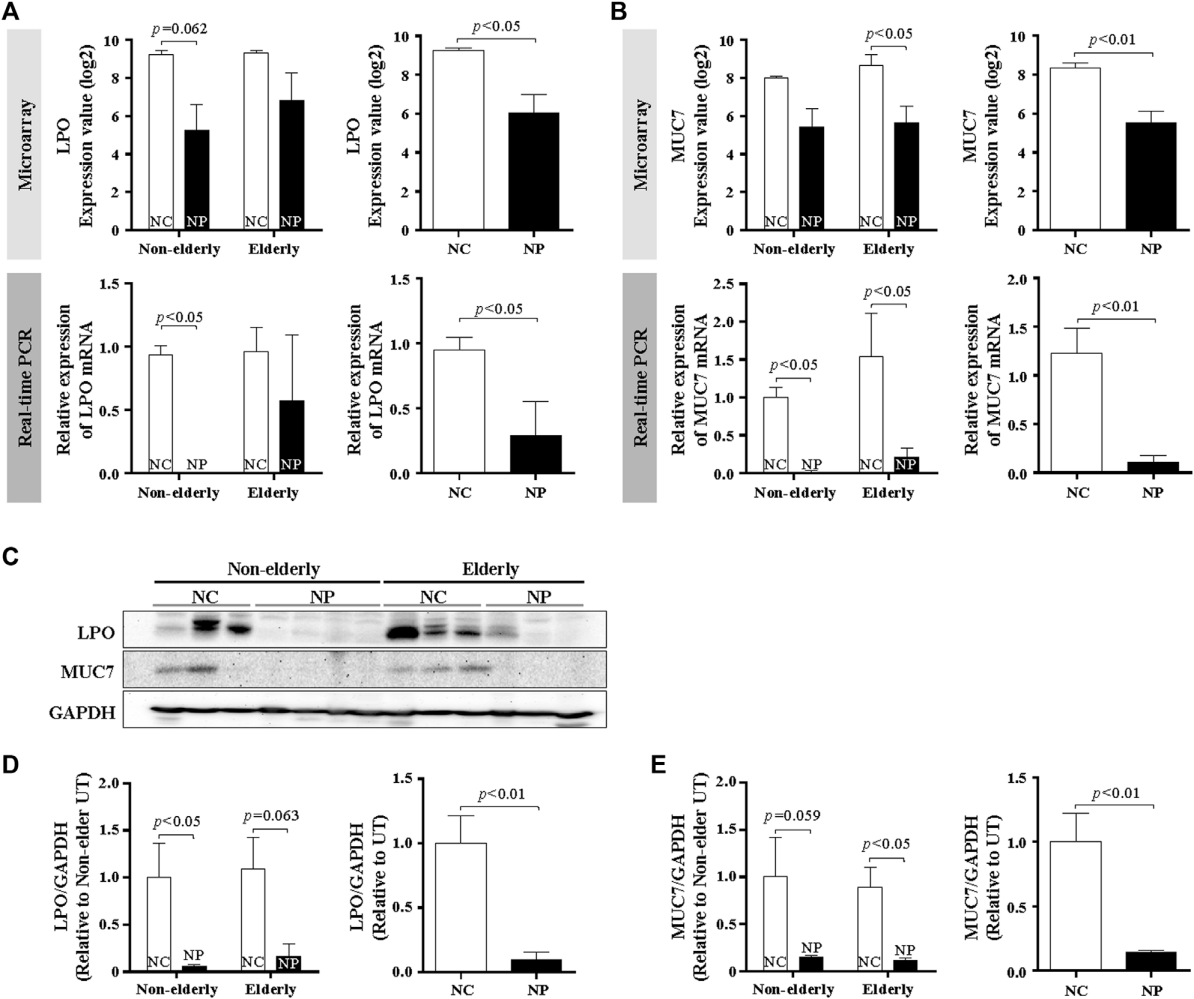
MUC7 and LPO expression in the sinonasal tissues from subjects with NP and normal controls **(A,B)** Validation of mRNA expression of selected genes, LPO, and MUC7 from microarray data by real-time PCR. Data are expressed as the means ± SE (NC versus NP, *n* = 16, LPO and MUC7, *p* < 0.05 and *p* < 0.01, respectively). **(C)** Validation of results using Western blot analysis of tissue extracts from NP and normal control subjects. Representative Western blots for each antibody are shown. **(D,E)** Quantification of band intensities for each represented blot was performed using ImageJ. Data are expressed as the means ± SE (NC versus NP, *n* = 13, *p* < 0.01). NC, normal control; NP, nasal polyp.

**FIGURE 4:**
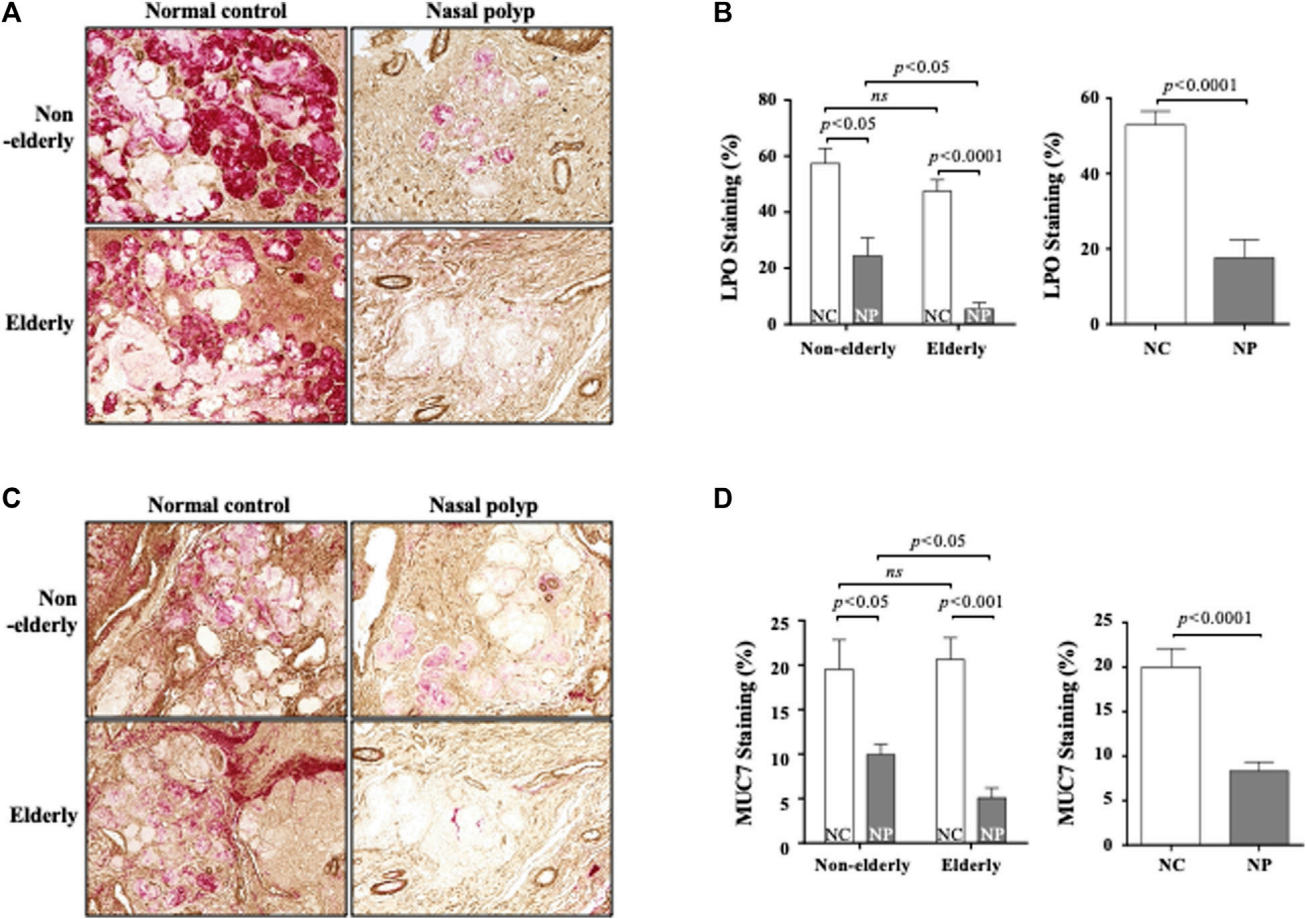
Immunohistochemistry of LPO and MUC7 in the sinonasal tissues from subjects with NP and normal controls. Data show representative images of LPO **(A)** and MUC7 **(C)** immunohistochemistry staining (red color). Each staining was double stained with αSMA for clarifying glandular structure (brown color). Original magnification ×100. Quantification of LPO **(B)** or MUC7 **(D)**-stained integrated density (%) per high power field of each group. Data are expressed as the means ± SE (non-elderly NP versus elderly NP, *n* = 15, *p* < 0.05; NC versus NP, *n* = 33, *p* < 0.0001, respectively) **(B,D)**. NC, normal control; NP, nasal polyp.

Serous cells are an important source of AMP. Significant reduction of serous cell-derived AMP gene expression in subjects with NP shown in microarray is consistent with the histologic changes in NP. The expression pattern of LPO and MUC7 by qPCR was evaluated to validate the microarray data related to serous cell-derived AMP. Consistent with their microarrays pattern, the mRNA expression of LPO and MUC7 was significantly reduced in qRT-PCR in NP compared with normal controls, regardless age (Figures 3A,B, *n* = 16, *p* < 0.05 and *p* < 0.01, respectively). The protein expression of LPO (Figures 3C,D) and MUC7 (Figures 3C,E) measured by Western blot also was significantly reduced in NP subjects versus normal controls, regardless of age (Figures 3D,E, *n* = 13, *p* < 0.01). The sinonasal tissues were examined for protein expression of LPO and MUC7 with immunohistochemistry (IHC) staining. LPO and MUC7-positive areas were significantly reduced in NP compared to control tissues regardless of age (Figures 4A–D, *n* = 33, *p* < 0.0001). However, there was no significant differences in LPO and MUC7-positive area between elderly control tissue and the non-elderly one. On the other hand, LPO and MUC7-positive areas were significantly reduced in elderly NP compared to the non-elderly NP (Figures 4B,D, *n* = 15, *p* < 0.05).

### Age-Related Regulations of Fibrinolytic System in Nasal Polyps Subjects and Normal Controls

**FIGURE 5:**
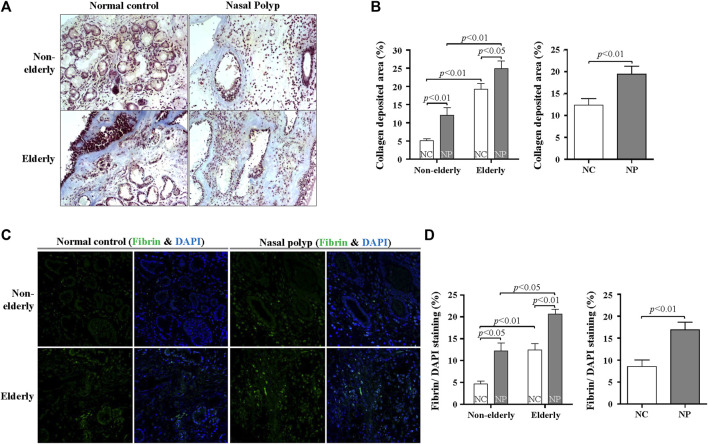
Age-related difference in collagen and fibrin deposition in subjects with NP and normal controls. **(A)** Masson’s trichrome staining was conducted to evaluate collagen deposition (blue color). Original magnification, ×200. **(B)** Quantification of trichrome-stained area (%) per high power field of each group. Data are expressed as the means ± SE (non-elderly NC versus elderly NC, *n* = 10, *p* < 0.01; non-elderly NP versus elderly NP, *n* = 10, *p* < 0.01; NC versus NP, *n* = 20, *p* < 0.01) **(C)** Representative immunofluorescence images stained with fibrin (green) and DAPI (blue). Original magnification: ×400. **(D)** Quantification of the fluorescence area (%) of fibrin per DAPI-stained nuclei. Data are expressed as the means ± SE (non-elderly NC versus elderly NC, *n* = 10, *p* < 0.01; non-elderly NP versus elderly NP, *n* = 10, *p* < 0.05; NC versus NP, *n* = 20, *p* < 0.01). NC, normal control; NP, nasal polyp.

**FIGURE 6:**
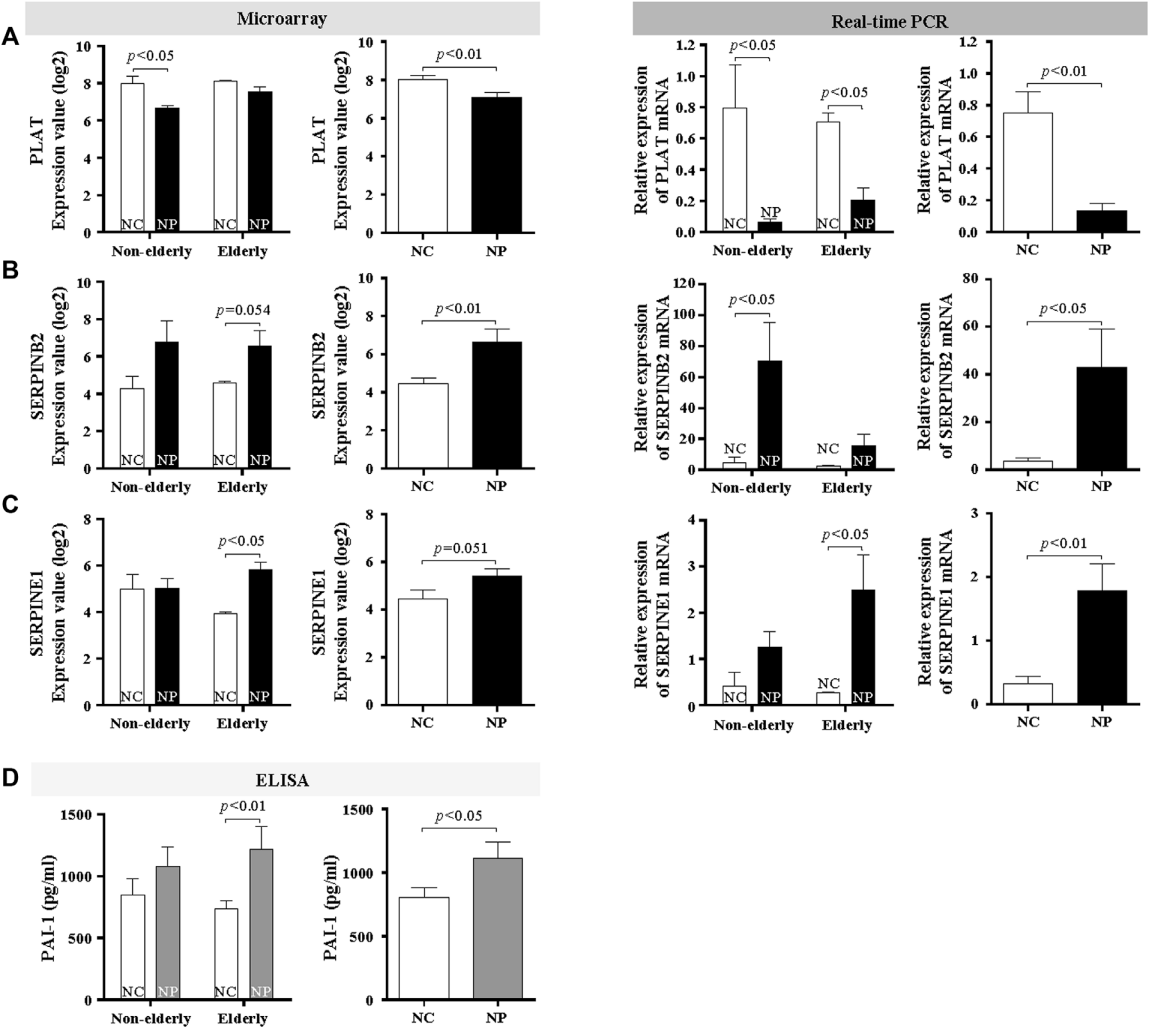
Age-related difference in collagen and fibrin deposition in subjects with NP and normal controls. Microarray data and validation of mRNA expression of fibrinolytic system genes by real-time PCR **(A)** PLAT (Data are expressed as the means ± SE; NC versus NP, *n* = 16, *p* < 0.01), **(B)** SERPINB2 (Data are expressed as the means ± SE; Non-elderly NC versus Non-elderly NP, *n* = 8, *p* < 0.01), and **(C)** SERPINE1 (Data are expressed as the means ± SE; Elderly NC versus Elderly NP, *n* = 8, *p* < 0.01). **(D)** PAI-1 protein levels were assessed by ELISA. Data are expressed as the means ± SE (Elderly NC vs. Elderly NP, *n* = 15, *p* < 0.01). NC, normal control; NP, nasal polyp.

Other tissue remodeling-related genes that are significantly upregulated in microarray were fibrinolysis system-related genes. Therefore, trichrome staining for collagen deposition and fibrin IHC staining was performed. Collagen deposition was increased with age in normal controls (Figures 5A,B, *n* = 10, *p* < 0.01) Collagen deposition was even more pronounced in the elderly NP compared with elderly controls (Figures 5A,B, *n* = 10, *p* < 0.05). Overall collagen deposition in NP tissues was higher than that of overall normal controls (n = 20, *p* < 0.01). Immunofluorescence was performed to determine fibrin deposition. In normal controls, there was a significant age-related increase in fibrin deposition (Figures 5C,D, *n* = 10, *p* < 0.01). A remarkable increase in fibrin deposition is present in overall NP group versus normal controls (Figure 5D, *n* = 20, *p* < 0.01), with a higher expression in elderly NP subjects versus the non-elderly NP group (Figure 5D, *n* = 10, *p* < 0.05). Regulation of fibrinolytic system genes was investigated by qRT-PCR to confirm the microarray results. There was reduced mRNA expression of PLAT (tPA gene) in NP subjects versus controls regardless of age (Figure 6A, *n* = 16, *p* < 0.01). There was significantly increased expression of SERPINB2 (PAI-2 gene) in non-elderly NP versus non-elderly controls (Figure 6B, *n* = 8, *p* < 0.05) and elevated expression of SERPINE1 (PAI-1 gene) primarily in elderly NP versus elderly controls (Figure 6C, *n* = 8, *p* < 0.05). PAI-1 is known to play the main inhibitor of tPA and uPA. Therefore, further investigation of PAI-1 protein production was conducted by ELISA using tissue extracts from NP subjects and normal controls. Consistent with qRT-PCR findings, PAI-1 protein production was significantly higher in elderly NP versus elderly controls (Figure 6D, *n* = 15, *p* < 0.01). PAI-1 protein levels were also significantly elevated in overall NP group compared with normal controls (Figure 6D right column, *n* = 39, *p* < 0.01).

## Discussion

This study explored age-related differential gene regulation by investigating microarray data and confirmed the differential regulation by qRT-PCR, histologic evaluation, Western blots, and ELISA. Using untargeted global gene expression profiling, 55 upregulated and 223 downregulated genes were found in NP subjects versus normal controls. Age-related differences in gene expression of NP and normal controls were investigated by dividing the study population into non-elderly and elderly subjects. We made a 15 years age gap to make a clear distinction between the two age groups. The signature genes include antimicrobial activity (BPIFB3, BPIFB2, LPO, and MUC7) (Sharma et al., 1998; Leclair, 2003; Plager et al., 2010; Widdicombe and Wine, 2015; Ma et al., 2018), fibrinolytic system (SERPINB2 and PLAT) (Takabayashi et al., 2013; Kim et al., 2015; Mueller et al., 2019) and the T2- related inflammation (POSTN) (Plager et al., 2010; Kim et al., 2017) and these genes are consistent with previous published reports. CEACAM5 gene expression is highly upregulated (10.24 fold) in NP versus normal controls, especially in elderly NP subjects versus elderly normal controls (12.23 fold). CEACAM5 mediates bacterial adhesion and exploits their receptors’ immunosuppressive function to bind pathogens to colonize mucosal surface (Hill et al., 2005; Min et al., 2012). Significantly reduction of CEACAM5 in the elderly NP may play role in decreased innate immune function in the elderly with NP. BPIFB2 and LPO, known as serous-derived AMP, were downregulated in non-elderly NP versus non-elderly normal controls. In addition, the fibrinolytic system-related gene (SERPINE1) were upregulated in elderly NP subjects versus elderly normal controls. In our microarray, tissue remodeling-related genes were most commonly shown as differentially regulated genes. In addition, our previous studies showed striking histologic differences between NP tissues and normal controls in tissue remodeling around submandibular glands. We also observed age-related differences in tissue remodeling, such as tissue fibrosis and glandular structural changes. Therefore we focused on genes related to tissue remodeling such as LPO, MUC7, and PAI-1 in this study.

One major aspect of the microarray results was differential regulation of genes related to SMG remodeling, present in chronic rhinosinusitis (Majima et al., 1997). SMG produce fluid, mucus, and antimicrobial molecules to maintain the host defense system. Loss of expression of antimicrobial activity-related genes may reflect the reduction of serous cells in SMG. GO analysis results of down-regulated genes between NP versus normal controls indicate that mammary gland development in biological processes and antimicrobial response in protein class showed the highest fold enrichment (Supplementary Figure SE2). Consistent with GO results, histological results showed structural changes in SMG, such as decreased glandular cells and glandular cell metaplasia in elderly controls and NP. Subsequently, it was found that reduced LPO and MUC7 expression were related to NP regardless of age through qRT-PCR, Western blot, and IHC. Interestingly, there was significant reduction of LPO and MUC7-positive staining area in elderly NP compared to non-elderly NP. This result suggests that there may be further decrease of serous cell function in elderly NP compared to young NP. These results suggest that serous cells, as a source of AMP, are essential for airway defense. Serous cell loss may cause structural breakdown of nasal mucosa and functional consequence of reduced AMP, leading to more bacterial colonization. However, the mechanism of SMG in NP is unclear and merits further exploration.

Another result indicates that the expression of fibrinolytic system-related genes differs with age. Increased collagen and fibrin deposition was found in elderly normal controls compared with non-elderly controls, and more pronounced in elderly NP subjects. Previously published reports show increased fibrin deposition in NP, although collagen deposition in CRS with NP would be less pronounced compared to that in CRS without NP (Molet et al., 2003; Takabayashi et al., 2013; Takabayashi and Schleimer, 2020). This study show increased fibrin and collagen deposition in elderly controls and elderly NP subjects, which was not previously reported. The results of qRT-PCR reveal that PLAT (t-PA gene) expression is decreased and SERINB2 (PAI-2 gene) increased in NP, regardless of age, but SERPINE1 (PAI-1 gene) is increased in elderly NP, suggesting the importance of PAI-1 in the pathogenesis of NP in the elderly. Previous studies report that elevated SERPINE1 levels are correlated with cellular senescence and the aging process (Yamamoto et al., 2005; Kortlever et al., 2006; Eren et al., 2014). We have been studying the role of PAI-1 in the airway remodeling of asthma (Cho et al., 2001; Jo et al., 2018; Cho et al., 2019). However, it has not been known about the role of PAI-1 in NP, especially in age-related roles. This study shows that PAI-1 may play a role in the pathogenesis of NP, especially in the elderly population, and PAI-1 can be a potential treatment target in elderly NP.

This study is not a clinical study with a large cohort but a mechanistic study with a small number of subjects. Therefore the findings in this study need to be confirmed in a larger cohort. This study investigated the age-related differences in patients with CRSwNP and normal controls but not in patients with CRSsNP. It would be interesting to see age-related differential regulation of genes in patients with CRSsNP.

In summary, this study demonstrates that NP exhibit a reduction of SMG-derived antimicrobial genes and increased production of fibrogenesis related genes. Histological analysis showed an age-related reduction of SMG in normal controls, and further decrease in NP subjects. The gene expression and protein production of PAI-1 was significantly elevated in elderly NP compared with elderly normal controls, which is consistent with increased collagen and fibrin deposition in this age group. These results may provide valuable information for age-related treatment targets in CRSwNP.

## Data Availability

The datasets presented in this study can be found in online repositories. The names of the repository/repositories and accession number(s) can be found below: “GEO with accession GSE194282 https://www.ncbi.nlm.nih.gov/geo/query/acc.cgi?acc=GSE194282.”

## Abbreviations

AMP, antimicrobial peptides and proteins; BPIF, BPI fold-containing family; CRS, Chronic rhinosinusitis; CRSsNP, CRS without nasal polyps; CRSwNP, CRS with nasal polyps; DEG, differentially expressed gene; GO, Gene Ontology; IPA, Ingenuity^®^ Pathway Analysis; LPO, Lactoperoxidase; PAI, plasminogen activator inhibitor; PAS, periodic acid-Schiff; PLUNC, palate, lung, and nasal epithelium carcinoma associated proteins; qPCR, Real-time PCR; SMG, Submucosal glands; t-PA, tissue-type plasminogen activator; u-PA, urokinase-type plasminogen activator.
