# Supplementary figures and images for: Age-Related Increase of Collagen/Fibrin Deposition and High PAI-1 Production in Human Nasal Polyps
Source: Front Pharmacol. 2022 May 31;13:845324. doi: 10.3389/fphar.2022.845324 (PMC9193225; doi:10.3389/fphar.2022.845324)

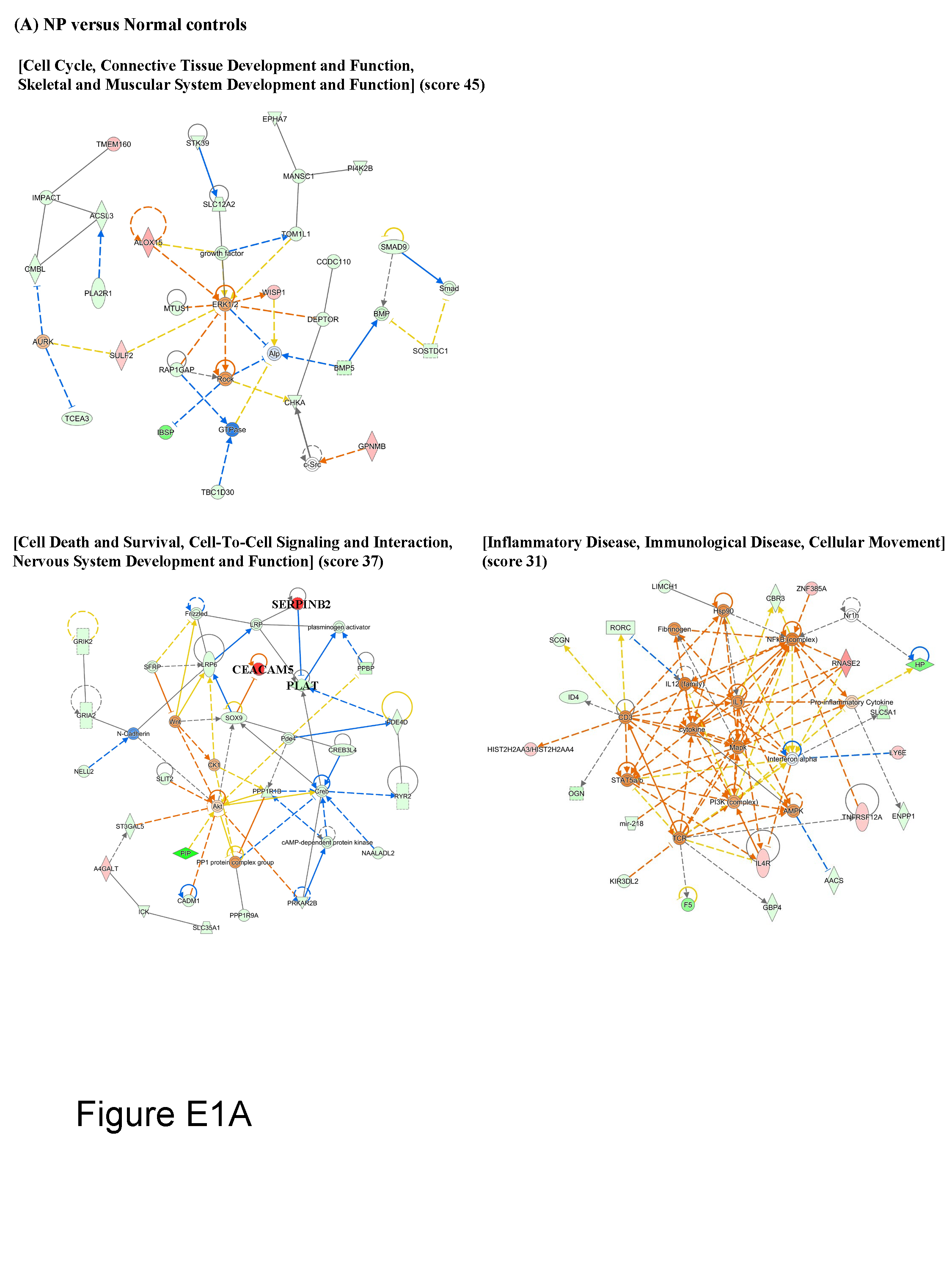

Supplement: Supplementary file 1 [file DataSheet2.zip › Figure E1A.TIFF]

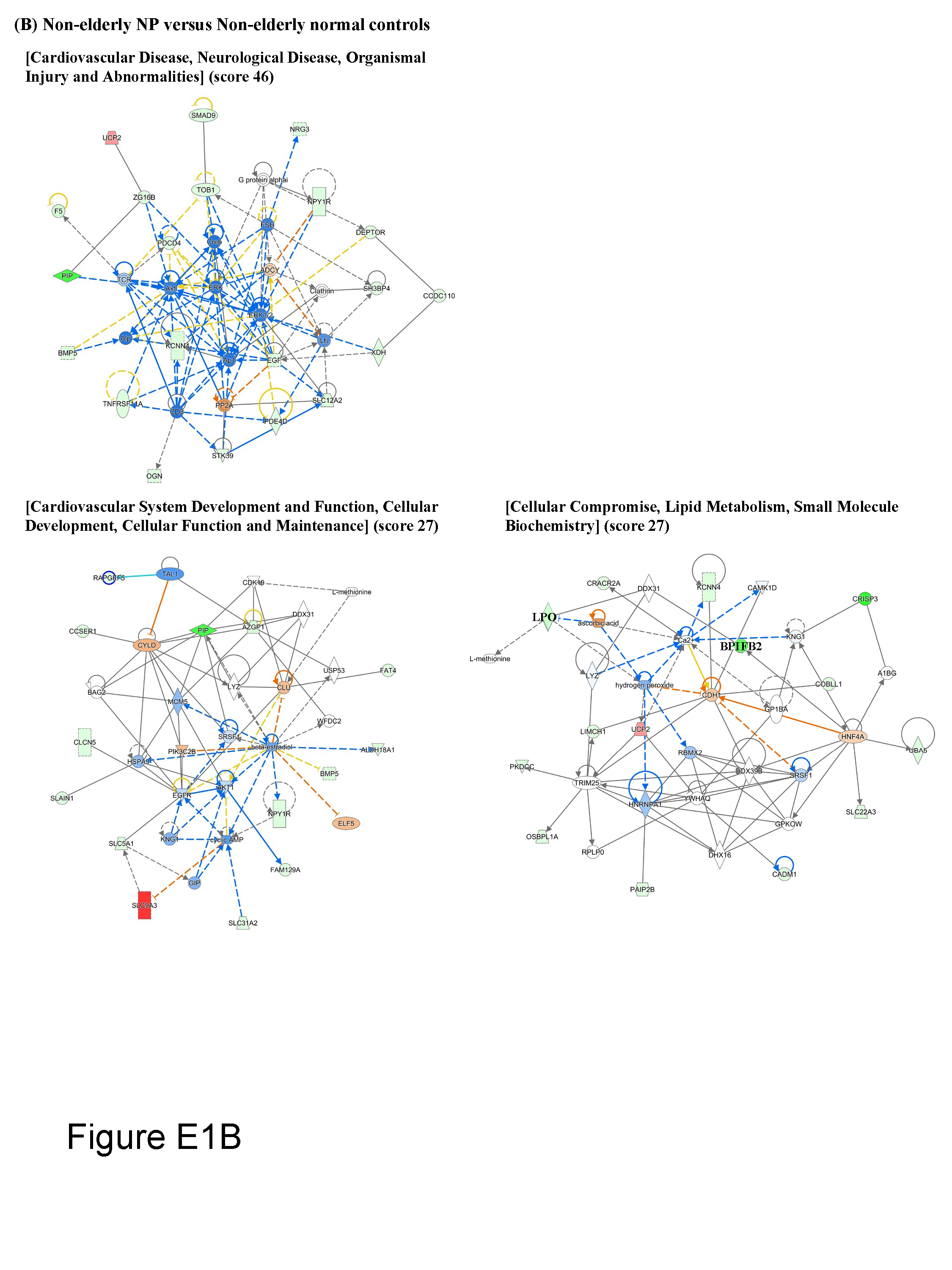

Supplement: Supplementary file 1 [file DataSheet2.zip › Figure E1B.TIFF]

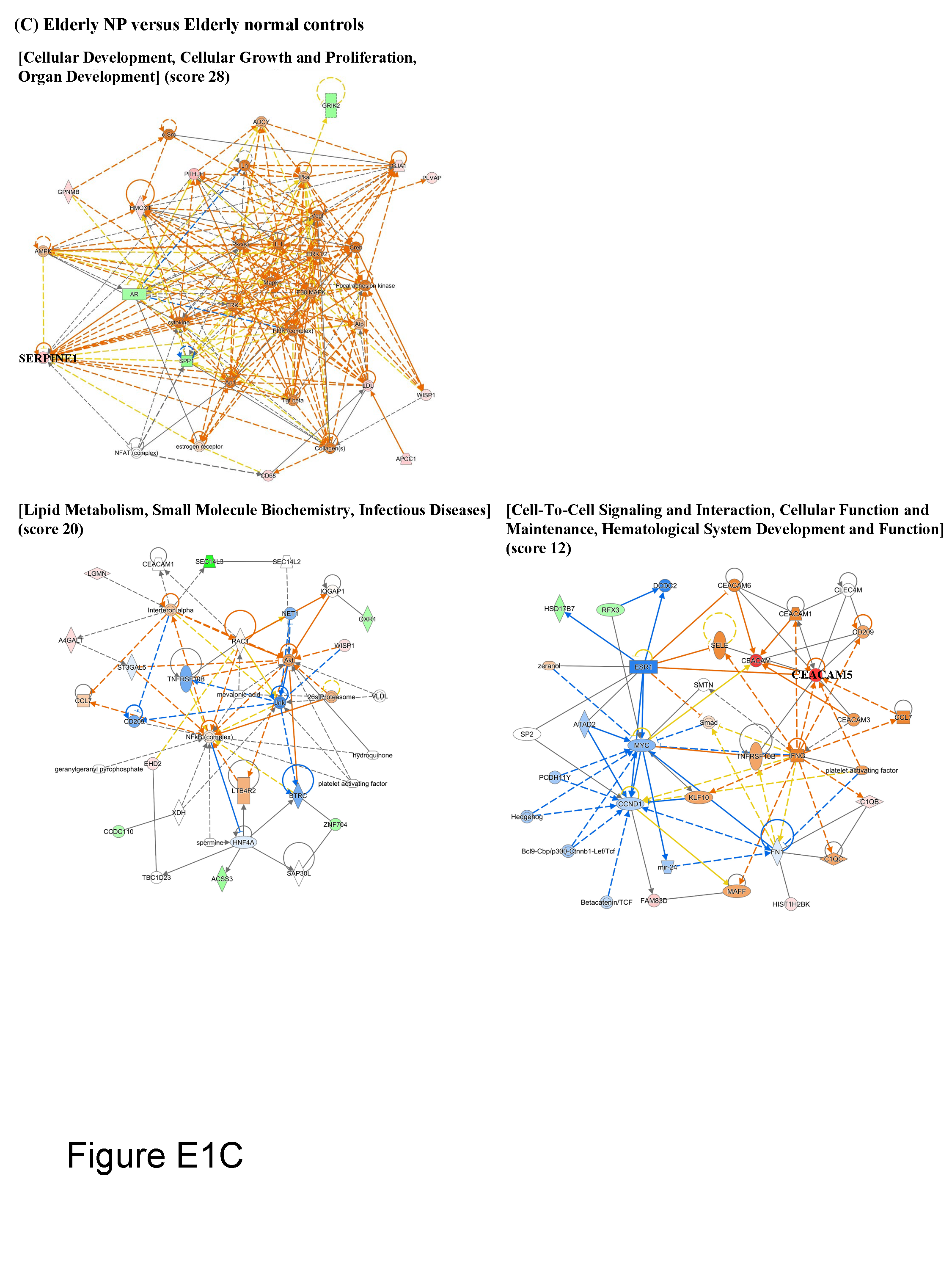

Supplement: Supplementary file 1 [file DataSheet2.zip › Figure E1C.TIFF]

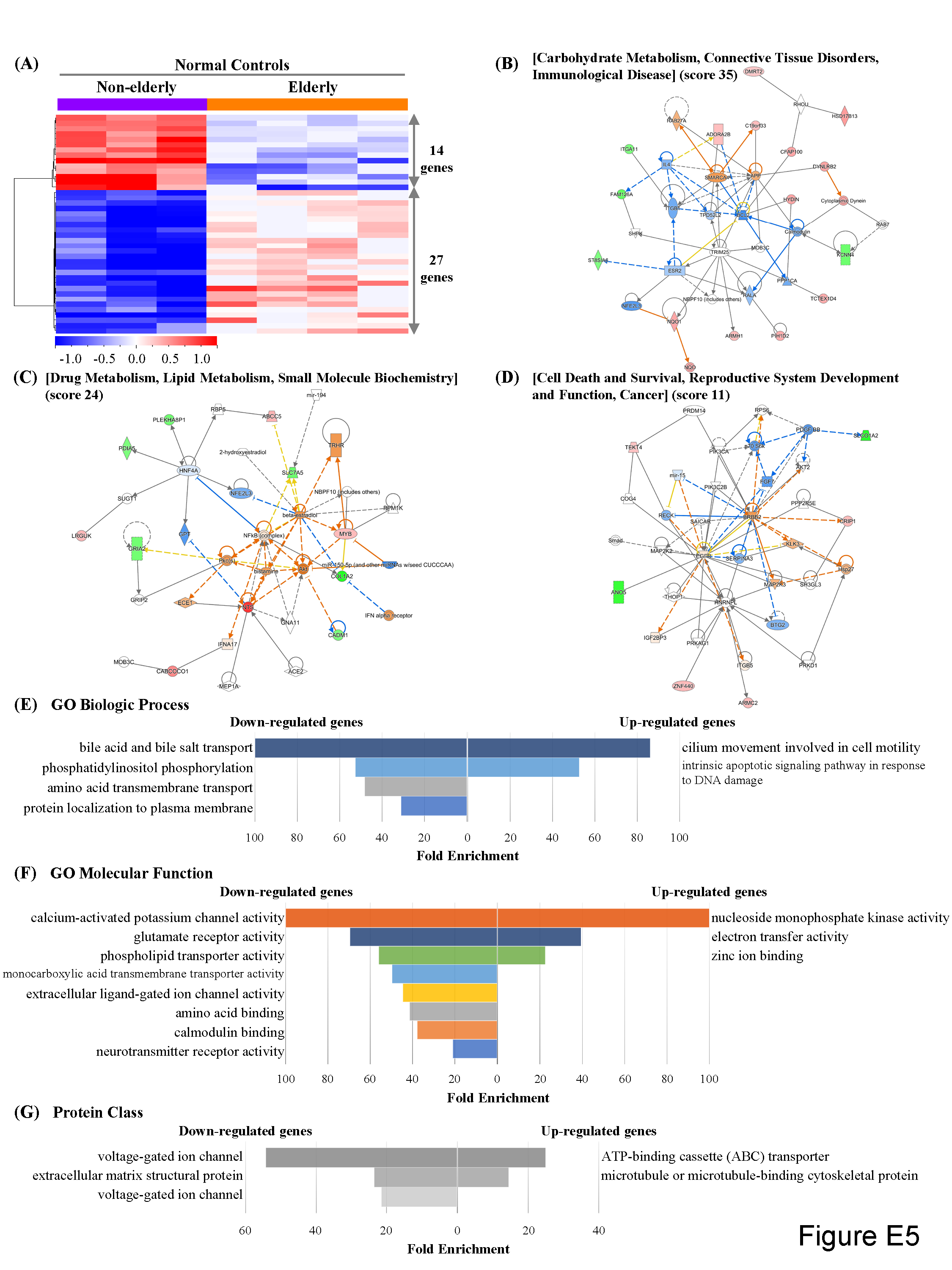

Supplement: Supplementary file 1 [file DataSheet2.zip › Figure E5.TIFF]
