# Supplementary material for: Age-Related Increase of Collagen/Fibrin Deposition and High PAI-1 Production in Human Nasal Polyps
Source: Front Pharmacol. 2022 May 31;13:845324. doi: 10.3389/fphar.2022.845324 (PMC9193225; doi:10.3389/fphar.2022.845324)
Supplement: Supplementary file 2 [file DataSheet1.docx]

**SUPPLEMENTARY MATERIAL**

**SUPPLEMENTAL METHODS**

**Bioinformatics analysis**

The commercial QIAGEN Ingenuity® Pathway Analysis (IPA®, QIAGEN Redwood City, www.qiagen.com/ingenuity) software was used for gene interaction network and upstream regulatory analysis of differentially expressed genes (DEGs) identified in this study. Networks and appropriate scores of these genes were algorithmically generated based on their connectivity. Each score is the numerical value used to rank a network according to how relevant this network is to the original list of focus genes in the Ingenuity Knowledge Base. It does not indicate the quality or significance of the network. Upstream transcriptional regulators were presented based upon the number of known targets of these regulators that exist. An overlap *p*-value was measured by significant overlap between genes in the data set and known targets regulated by the transcriptional regulator.

Gene Ontology (GO) overrepresentation analyses, including biological process, molecular function, and protein class, were conducted using GO Consortium (http://geneontology.org/) and PANTHER (http://pantherdb.org/)(1). The analysis was performed using Fisher’s Exact test with Bonferroni correction (*p*< 0.05). Fold enrichment of GO term was obtained by comparing it to the background frequency of the total genes annotated to the number of input genes corresponding to the same term. Results were presented as highest to lowest fold enrichment.

**SUPPLEMENTAL RESULTS**

**Gene ontology and network analysis in NP versus normal controls**

The 278 differentially expressed genes between normal controls and NP subjects were analyzed to identify gene networks by IPA software; 23 significant networks were obtained with a score between 2 and 45. The top three gene networks are associated with cell cycle, connective tissue development and function, skeletal and muscular system development and function (score 45), cell death and survival, cell-to-cell signaling and interaction, nervous system development and function (score 37), inflammatory disease, immunological disease, cellular movement (score 31) (Figure E1A). The top-ranked five upstream transcriptional regulators are presented in Table E3.

The GO analysis results between NP and normal subjects show that the upregulated genes are significantly enriched in the biological process, including kidney development, positive regulation of extrinsic apoptotic signaling pathway, and osteoblast differentiation. The down-regulated genes are significantly enriched in biological processes, including mammary gland development, iron ion transmembrane transport, and oligodendrocyte differentiation (Figure E2A). The result show that the upregulated genes are significantly enriched in inositol phosphate phosphatase activity, acetylcholine receptor regulator activity, and peptide hormone receptor binding according to the overrepresented GO molecular function. The down-regulated genes are significantly enriched in UDP-galactose transmembrane transporter activity, calcium-activated potassium channel activity, and myosin binding (Figure E2B). Additionally, the GO protein class analysis reveals that the upregulated genes are involved in the endodeoxyribonuclease, histone, and growth factors. The down-regulated genes are involved in the antimicrobial response protein, cadherin, and transaminase (Figure E2C).

**Gene ontology and network analysis in non-elderly NP versus non-elderly normal controls**

The top-ranked three gene networks are associated with cardiovascular disease, neurological disease, organismal injury and abnormalities (score 46), cardiovascular system development and function, cellular development, cellular function and maintenance (score 27) and cellular compromise, lipid metabolism, small molecule biochemistry (score 27) (Figure E1B). The top-ranked five upstream transcriptional regulators are presented in Table E3.

The GO analysis results between non-elderly NP versus non-elderly controls shows that upregulated genes are significantly enriched in the biological process, including sodium ion transmembrane transport, mitochondrial transmembrane transport, and regulation of intracellular pH. The down-regulated genes are significantly enriched in biological processes, including stress fiber assembly, cellular copper ion homeostasis, and acetylcholine receptor signaling pathway (Figure E3A). The results show that the upregulated genes are significantly enriched in acetylcholine binding, acetylcholine receptor activity, and solute-proton antiporter activity according to the overrepresented GO molecular function. The down-regulated genes are significantly enriched in UDP-glucosyltransferase activity, calcium-activated potassium channel activity, and sodium-chloride symporter activity (Figure E3B). Additionally, the GO protein class analysis reveals that upregulated genes are involved in secondary carrier transporter, and down-regulated genes in phosphatase inhibitor, growth factor, and guanyl-nucleotide exchange factor (Figure E3C).

**Gene ontology and network analysis in elderly NP versus elderly normal controls**

The top-ranked three gene networks are associated with cellular development, cellular growth and proliferation, organ development (score 28), lipid metabolism, small molecule biochemistry, infectious diseases (score 20) and cell-to-cell signaling and interaction, cellular function and maintenance, hematological system development and function (score 12) (Figure E1C). The top-ranked five upstream transcriptional regulators are presented in Table E3.

The GO analysis results between elderly NP versus elderly normal controls shows that upregulated genes are significantly enriched in the biological process, including heme metabolic process, vacuolar protein processing, and osteoblast differentiation. Downregulated genes are significantly enriched in biological processes, including stress synaptic transmission, glutamatergic, and modulation of chemical synaptic transmission (Figure E4A). According to the GO molecular function, upregulated genes are significantly enriched in peptide hormone receptor-binding, heparin-binding, and integrin-binding. Downregulated genes are significantly enriched in glutamate receptor activity, extracellular ligand-gated ion channel activity, and amino acid binding (Figure E4B). Additionally, the GO protein class analysis reveals that upregulated genes are involved in gap junction, apolipoprotein, and membrane-bound signaling molecule. Downregulated genes are involved in reductase, ligase, and cytokine (Figure E4C).

**Differential gene expression analysis between elderly vs non-elderly normal controls**

Differential gene expression between elderly and non-elderly subjects were investigated. In the comparison of the DEG between elderly and non-elderly, 41 significant genes were identified. Fourteen genes (66.0%) are upregulated, and 27 (34.0%) down-regulated (Figure E5A). Among the DEGs, the top 10 upregulated and downregulated genes and top-ranked five upstream transcriptional regulators are listed in Table E3. One of the target molecules, NQO1, is upregulated as a part of the oxidative stress response. The top-ranked three gene networks that are associated include carbohydrate metabolism, connective tissue disorders, immunological disease (score 35, Figure E5B), drug metabolism, lipid metabolism, small molecule biochemistry (score 24, Figure E5C) and cell death and survival, reproductive system development and function, cancer (score 11, Figure E5D).

The GO analysis results show that upregulated genes are enriched in the biological process, cilium movement involved in cell motility, and intrinsic apoptotic signaling pathway in response to DNA damage (Figure E5E). The GO molecular function and GO protein class analysis are presented in Figure E5F and G.

**REFERENCE**

1. Mi H, Muruganujan A, Huang X, Ebert D, Mills C, Guo X, Thomas PD. Protocol update for large-scale genome and gene function analysis with the panther classification system (v.14.0). *Nat Protoc* 2019;14(3):703-721.

**Table E1. Subject characteristics of nasal polyp subjects and normal controls**

| Characteristics | Normal Control (n=22) | Nasal Polyps (n=20) |
| --- | --- | --- |
| Age |  |  |
| Mean ± SD | 53.6 ± 17.9 | 44.8 ± 19.0 |
| Median (range) | 53 (27 - 89) | 39 (22 - 81) |
| Sex, n (%) |  |  |
| Male | 7 (31.8%) | 9 (45%) |
| Female | 15 (68.2%) | 11 (55%) |
| Race, n (%) |  |  |
| White | 14 (63.6%) | 13 (65%) |
| Black or African American | 5 (22.7%) | 4 (20%) |
| Asian | 1 (4.5%) | 1 (5%) |
| Others | 2 (9.2%) | 2 (10%) |
| Atopy, n (%)* | **1 (4.5%)** | **6 (30%)** |
| Asthma, n (%)** | **3 (13.6%)** | **9 (45%)** |

**Fisher's exact test *Atopy p=0.0408, **Asthma p=0.04**

**Table E2. Subject characteristics of nasal polyp subjects and normal controls by age**

| **Characteristic**  **(microarray)** | | **Normal controls** | | **Nasal polyps** | |
| --- | --- | --- | --- | --- | --- |
|  |  | **Non-elderly**  **(18-49)** | **Elderly**  **(≥65)** | **Non-elderly**  **(18-49)** | **Elderly**  **(≥65)** |
| **Total no. of subjects** | | n=3 (1M/2F) | n=4 (1M/3F) | n=3 (2M/1F) | n=4 (4M) |
| **Age range (Median)** | | 22-47 (28.0) | 66-72 (69.5) | 37-40 (37.0) | 65-70 (67.0) |
| **Characteristic**  **(Real-time PCR)** | | **Normal controls** | | **Nasal polyps** | |
|  |  | **Non-elderly**  **(18-49)** | **Elderly**  **(≥65)** | **Non-elderly**  **(18-49)** | **Elderly**  **(≥65)** |
| **Total no. of subjects** | | n=4 (2M/ 2F) | n=4 (1M/ 3F) | n=4 (3M/ 1F) | n=4 (4M) |
| **Age range (Median)** | | 22-47 (31.5) | 66-72 (69.5) | 34-40 (37.0) | 65-70 (67.0) |
| **Characteristic**  **(Staining & Western blot)** | | **Normal controls** | | **Nasal polyps** | |
|  |  | **Non-elderly**  **(18-49)** | **Elderly**  **(≥65)** | **Non-elderly**  **(18-49)** | **Elderly**  **(≥64)** |
| **PAS staining** | **Total no. of subjects** | n= 15 | n= 4 | n= 15 | n= 14 |
|  | **Age range (Median)** | 19-49 (41.0) | 66-73 (69.5) | 23-48 (38.0) | 66-75 (69.5) |
| **IHC Staining** | **Total no. of subjects** | n= 10 | n= 8 | n= 10 | n= 5 |
|  | **Age range (Median)** | 27-46 (40.5) | 65-89 (67) | 24-42 (33.5) | 64-81 (73) |
| **Trichrome**  **& IF staining** | **Total no. of subjects** | n= 5 | n= 5 | n= 5 | n= 5 |
|  | **Age range (Median)** | 25-45 (41.0) | 66-73 (68.0) | 19-45 (27.0) | 70-72 (71.0) |
| **Western blot** | **Total no. of subjects** | n= 3 | n= 3 | n= 4 | n= 3 |
|  | **Age range (Median)** | 27-44 (43.0) | 68-89 (74.0) | 24-44 (36.0) | 64-76 (73.0) |
| **Characteristic**  **(ELISA)** | | **Normal controls** | | **Nasal polyps** | |
|  |  | **Non-elderly**  **(18-49)** | **Elderly**  **(≥60)** | **Non-elderly**  **(18-49)** | **Elderly**  **(≥60)** |
| **ELISA** | **Total no. of subjects** | n= 11 | n= 10 | n= 13 | n= 5 |
|  | **Age range (Median)** | 27-46  (43.0) | 60-89  (65.5) | 24-47  (38.0) | 64-81  (76.0) |

**Table E3. Top5 upstream transcriptional regulators using Ingenuity Pathway Analysis (IPA)**

| **Nasal polyps versus Normal controls** | | |
| --- | --- | --- |
| **Top 5 upstream transcriptional regulators** | ***p*-value of overlap** | **Target molecules in dataset** |
| NKX2-3 | 3.80E-04 | CD68,FAM198B,FLRT2,GMPR,HIST2H2AA3/HIST2H2AA4,  LINC01235, LY6E,MAMDC2,POSTN,ZNF704 |
| ETV1 | 2.30E-03 | ACSL3,HSD17B7,SOX9 |
| FOXA1 | 5.38E-03 | ALDH6A1,PAM,RORC,SOX9,TNFRSF12A |
| HIC1 | 7.63E-03 | CA2,CYP7B1,ID4,SOX9 |
| JUN | 8.61E-03 | AR,CD68,GSTP1,MAOB,mir- 146,PTHLH,SERPINB2,SULF2, ZNF385A |
| **Non-elderly nasal polyps versus Non-elderly normal controls** | | |
| **Top 5 upstream transcriptional regulators** | ***p*-value of overlap** | **Target molecules in dataset** |
| SATB1 | 4.32E-03 | F5,FAM129A,GPT2 |
| NANOG | 5.85E-03 | PDCD4,RAPGEF5,TOB1 |
| NCOR1 | 1.31E-02 | AZGP1,STK39 |
| GRIP1 | 1.36E-02 | GRIA2 |
| ATF4 | 1.84E-02 | TNFRSF11A,XDH |
| **Elderly nasal polyps versus Elderly normal controls** | | |
| **Top 5 upstream transcriptional regulators** | ***p*-value of overlap** | **Target molecules in dataset** |
| JUN | 5.31E-08 | AR,CD68,GJA1,HMOX1,PTHLH,SERPINE1,SPP1 |
| ING2 | 1.71E-06 | SERPINE1,SPP1 |
| KDM3A | 1.71E-05 | HMOX1,SERPINE1 |
| SMAD3 | 2.11E-05 | HMOX1,PTHLH,SERPINE1,SPP1 |
| ELK3 | 2.56E-05 | HMOX1,SERPINE1 |

**Table E4. List of the top 10 upregulated and downregulated genes and Top5 upstream transcriptional regulators in elderly versus non-elderly normal controls.**

| **Elderly normal controls versus Non-elderly normal controls** | | | | | | | | | | |
| --- | --- | --- | --- | --- | --- | --- | --- | --- | --- | --- |
| **Top 10 up-regulated genes** | **FDR** | **Fold Change** | | ***p*-value** | |  | **Top 10 down-regulated genes** | **FDR** | **Fold Change** | ***p*-value** |
| **NTS** | 0.031 | 5.72 | | 4.55E-04 | |  | **COL1A2** | 0.037 | -2.82 | 8.52E-04 |
| **C10orf107** | 0.037 | 3.34 | | 7.16E-04 | |  | **SLCO1A2** | 0.009 | -2.71 | 4.83E-05 |
| **BEST4** | 0.037 | 2.84 | | 8.73E-04 | |  | **ANO5** | 0.037 | -2.50 | 8.63E-04 |
| **HSD17B13** | 0.037 | 2.79 | | 6.80E-04 | |  | **CCDC129** | 0.034 | -2.15 | 5.12E-04 |
| **DYNLRB2** | 0.009 | 2.59 | | 4.69E-05 | |  | **FAM126A** | 0.028 | -1.98 | 3.42E-04 |
| **HYDIN** | 0.037 | 2.58 | | 6.88E-04 | |  | **SLC7A5** | 0.037 | -1.89 | 8.91E-04 |
| **CFAP100** | 0.020 | 2.53 | | 1.70E-04 | |  | **KCNN4** | 0.027 | -1.83 | 2.58E-04 |
| **NQO1** | 0.039 | 2.52 | | 9.66E-04 | |  | **PLEKHA8P1** | 0.031 | -1.84 | 4.22E-04 |
| **C10orf67** | 0.031 | 2.41 | | 4.52E-04 | |  | **GRIA2** | 0.037 | -1.78 | 8.08E-04 |
| **TCTEX1D4** | 0.009 | 2.4 | | 1.53E-05 | |  | **LINC01614** | 0.009 | -1.72 | 2.38E-05 |
| **Elderly normal controls versus Non-elderly normal controls** | | | | | | | | | | |
| **Top 5 upstream transcriptional regulators** | | | ***p*-value of overlap** | | **Target molecules in dataset** | | | | | |
| NFE2L3 | | | 1.78E-03 | | NQO1 | | | | | |
| ETS1 | | | 1.84E-03 | | COL1A2,MYB,NQO1 | | | | | |
| MAFG | | | 5.33E-03 | | NQO1 | | | | | |
| GRIP1 | | | 7.10E-03 | | GRIA2 | | | | | |
| MYB | | | 7.67E-03 | | COL1A2,MYB | | | | | |

**Figure E1. Top3 gene interaction networks using Ingenuity Pathway Analysis (IPA).** The intensity of the node color (red or green) indicates the degree of up-or down-regulated, respectively. Uncolored genes or molecules are the intermediate in the computationally generated networks based on the evidence but not found in our microarray data.

**Figure E2. Gene ontology (GO) results in nasal polyp subjects versus normal controls.** Each bar indicates the top 10 ranked by fold enrichment of the corresponding GO terms; (A) biological process, (B) molecular function, and (C) protein class.

**Figure E3. Gene ontology (GO) results in non-elderly NP versus non-elderly normal controls.** Each bar indicates the top 10 ranked by fold enrichment of the corresponding GO terms; (A) biological process, (B) molecular function, and (C) protein class.

**Figure E4. Gene ontology (GO) results in elderly NP versus elderly normal controls.** Each bar indicates the top 10 ranked by fold enrichment of the corresponding GO terms; (A) biological process, (B) molecular function, and (C) protein class.

**Figure E5. Differentially expressed genes and mechanisms in elderly versus non-elderly subjects with normal controls.** (A) The heatmaps represent expression profiles of the 41 differentially expressed genes in elderly and non-elderly normal controls. A red-blue color scale depicts expression levels (upregulated; red and down-regulated; blue). There were top three gene networks, classified as ‘carbohydrate metabolism, connective tissue disorders, immunological disease (score 35, B)’, ‘drug metabolism, lipid metabolism, small molecule biochemistry (score 24, C)’ and ‘cell death and survival, reproductive system development and function, cancer’ (score 11, D). The intensity of the node color (red or green) indicates the degree of up-or down-regulated, respectively. Uncolored genes or molecules are the intermediate in the computationally generated networks based on the evidence but not found in our microarray data. Each bar indicates the top 10 ranked by fold enrichment of the corresponding GO terms; (E) biological process, (F) molecular function, and (G) protein class.
